# PD-L1 Assessment in Needle Core Biopsies of Non-Small Cell Lung Cancer: Interpathologist Agreement and Potential Associated Histopathological Features

**DOI:** 10.5146/tjpath.2023.01609

**Published:** 2024-01-22

**Authors:** Ezgi Hacıhasanoglu, Buket Bambul Sıgırcı, Gamze Usul, Taha Cumhan Savlı

**Affiliations:** Department of Pathology, 1Yeditepe University, School of Medicine, İstanbul, Turkey; University of Health Sciences, Sisli Hamidiye Etfal Training Hospital, İstanbul, Turkey; Basaksehir Cam and Sakura City Hospital, İstanbul, Turkey; Istanbul Medipol University, School of Medicine, İstanbul, Turkey

**Keywords:** Programmed death ligand 1, Lung, Interobserver, Biopsy, Needle

## Abstract

***Objective: *** Immune checkpoint inhibitors are used in the treatment of non-small cell lung cancer (NSCLC). Programmed cell death-ligand 1 (PD-L1) immunohistochemistry (IHC) assessed by pathologists is subject to interobserver variability. In advanced/metastatic disease and inoperable patients, PD-L1 assessment relies on biopsy specimens, commonly needle core biopsies (NCB). We aimed to determine the interobserver agreement for PD-L1 tumor proportion score (TPS) in NSCLC NCBs and identify histopathological features that may be related to interobserver variability.

***Material and Methods:*** Sixty NSCLC NCBs with PD-L1 IHC were evaluated independently by four pathologists from different institutions. PD-L1 TPS was evaluated in three categories: no/low expression (<1%), intermediate expression (1%–49%), and high expression (≥50%). Histological tumor type, necrosis, tumor-infiltrating lymphocytes, tumor length/percentage in the biopsy, and crush/squeeze artifact was evaluated.

***Results: *** The statistical analysis of the three PD-L1 TPS categories demonstrated moderate agreement (Fleiss Kappa 0.477) in the no/low category, fair agreement (Fleiss Kappa 0.390) in the intermediate category, and almost perfect agreement (Fleiss Kappa 0.952) in the high category. A significant correlation (p=0.003) was found between the crush/squeeze artifact in NCB and rate of discordant TPS categories. There was no significant correlation between pathologists’ agreement in the TPS categories and histological tumor type, tumor length, tumor ratio, necrosis, and tumor-infiltrating lymphocytes.

***Conclusion:*** Our results demonstrated moderate agreement among pathologists for the PD-L1 TPS 1% cut-off in NSCLC NCB, which is lower than that reported in resection materials. The presence of crush/squeeze artifact in NCBs is significantly related to the rate of discordant TPS categories, suggesting that PD-L1 assessment of pulmonary NCBs requires an awareness of this artifact.

## INTRODUCTION

Lung cancer is the leading cause of death among cancers (1). The treatment of non-small cell lung cancer (NSCLC) has significantly advanced over the last two decades, contributing to an improvement in survival rates. Through the use of biomarkers-based treatment approaches, lung cancer patients have been able to receive personalized treatment. Recently, research on immune checkpoint inhibitors has made significant contributions to the development of novel treatment approaches for advanced-stage lung cancer. The evidence suggests that immunotherapy is superior to cytotoxic chemotherapy in a subset of patients, as well as the possibility of combining immunotherapy and chemotherapy (2).

Programmed cell death-ligand 1 (PD-L1) immunohistochemistry (IHC) is the first Food and Drug Administration (FDA)-approved companion diagnostic test for immune checkpoint inhibitors. The FDA approved the 22C3 assay as a companion diagnostic test for pembrolizumab, an immune checkpoint inhibitor. PD-L1 IHC is evaluated and scored by pathologists. Different scoring methods are employed in different organ tumors to examine the level of PD-L1 staining in tumor cells and/or inflammatory cells within the tumor stroma. To determine PD-L1 expression in NSCLC, the Tumor Proportion Score (TPS) is used, which is a percentage of viable tumor cells that stain partially or completely by PD-L1 at any intensity (3).

PD-L1 assessment is subject to variability between pathologists. This variability impacts the therapeutic outcome. The interobserver concordance of TPS in NSCLC has been investigated in a number of studies (4–11). The majority of these studies used resection material or tissue microarrays (TMA), while only one study included needle core biopsy (NCB) specimens. To the extent of our knowledge, no study has been published using only NCB materials (4–7,10). Image-guided transthoracic NCB is a routinely used diagnostic tool in lung masses, and the assessment of PD-L1 in advanced or metastatic disease and in inoperable patients is based on the least invasive biopsies. Therefore, it is imperative that NCB specimens be scored accurately in order to guide treatment decisions.

Among the histopathological factors that may affect interobserver agreement of PD-L1 scoring, only the histological type of the tumor was investigated in a previous study (8). Factors that may complicate the evaluation of PD-L1 in NSCLC NCBs, such as tumor necrosis, tumor-infiltrating lymphocytes, tumor length and percentage in the biopsy, and effect of crush/squeeze artifact have not been evaluated before.

The purpose of this study was to determine the interobserver agreement for PD-L1 TPS in NCB specimens of NSCLC and identify histopathological features of the tumor that may be related to interobserver variability.

## MATERIAL and METHODS

### Study Design and Case Selection

A search was conducted in the electronic database of a single center for patients diagnosed with NSCLC diagnosed through a NCB specimen. Cases with available PD-L1 immunohistochemistry (IHC) studies were documented. The study included 60 cases with at least 100 viable tumor cells on Hematoxylin-Eosin (HE) stained slides and PD-L1 positive tumor cells ranging from 0% to 100%. Representative HE slides and PD-L1 IHC slides of the cases were evaluated independently and blinded to the diagnosis by four pathologists from different institutions (EH, BBS, GU, TCS).

This study has been approved by the Ethics Committee for Non-Interventional Clinical Studies (decision number: 202211Y0307, date: 14/11/2022). This study protocol is in accordance with the Declaration of Helsinki of the World Medical Association.

### PD-L1 IHC Staining and Scoring

The PD-L1 IHC 22C3 pharmDx IHC assay (Agilent Technologies/Dako, Carpinteria, California, USA) was performed in all cases. All tissue samples underwent fixation in 10% neutral buffered formalin for 6-24 hours. Four-μm thick formalin-fixed, paraffin-embedded tissue sections were dried at 60°C for 30 minutes. PD-L1 IHC 22C3 pharmDx IHC assays were performed using an EnVision FLEX visualization system (Agilent, Santa Clara, USA) and an Autostainer Link 48 system (Dako). In accordance with the instructions of the manufacturer, positive and negative controls were used. All cases were stained within 6 months of sectioning.

**Figure 1:**
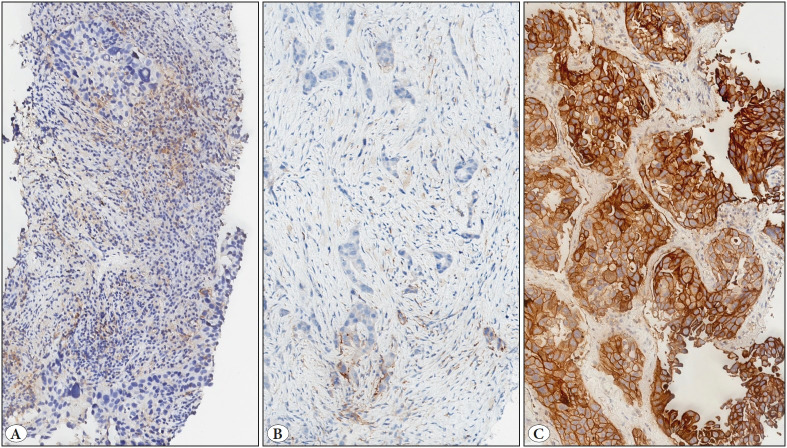
**A-C)** PD-L1 expression levels in tumor cells in NSCLC cases (x100). **A)** No/low expression (<1%) - Tumor cells are negative for PD-L1, while some tumor-associated immune cells show positive staining. **B)** Intermediate expression (1–49%) - A small proportion (1-10%) of the tumor cells show partial/complete membrane staining with PD-L1. **C)** High expression (≥50%) - Almost all tumor cells show membrane staining with PD-L1 at varying intensities.

The Tumor Proportion Score (TPS) is defined as the percentage of viable tumor cells with partial or complete PD-L1 membranous staining at any intensity (≥ 1+) relative to all viable tumor cells in the sample (3). The IHC results of 60 cases were evaluated by four pathologists and TPS was assigned by each. TPS was categorized into three expression levels: no/low expression (<1%), intermediate expression (1–49%) and high expression (≥50%) (Figure 1).

### Histopathological Parameters

A comprehensive examination of histopathological features that may be associated with interobserver variability was performed. The histological type of tumor was documented. Presence of necrosis was determined. Extent of tumor-infiltrating lymphocytes was evaluated as mild-moderate (<50%) or extensive (≥50%). Tumor length in the biopsy specimen was measured in each case. The percentage of tumor in the biopsy specimen was evaluated as <50% or ≥50%. Presence of crush/squeeze artifact was evaluated.

### Statistical Analysis

The descriptive statistics are expressed in terms of number (n) and percentage (%) for the variables in the study. Fleiss’s Kappa analysis was used to calculate the agreement between pathologists. Agreement for 1% and 50% cut-off values were assessed. Evaluation was done according to Landis and Koch’s methodology, according to the following: kappa value of 0-0.2 indicated slight agreement, 0.2-0.4 fair agreement, 0.4-0.6 moderate agreement, 0.6-0.8 substantial agreement, and 0.8-1 almost perfect agreement (12). Statistical analysis of quantitative independent data was conducted using the Mann-Whitney U test, and a qualitative analysis of independent data was conducted using the Chi-square test. The statistical significance level was taken as 5% in the calculations. The statistical analysis was carried out using IBM SPSS Statistics (version 26).

## RESULTS

### PD-L1 TPS Evaluation by Pathologists

Table 1 shows the number of cases scored by each pathologist as low, moderate, and high TPS (Table 1). In 31 cases, four pathologists scored within the same TPS category. In 29 cases, at least one pathologist scored at a different TPS category.

**Table 1:** Number of cases scored by each pathologist as low, moderate, and high TPS.

	**TPS**	**Number of cases (n)**	**Percentage (%)**
Observer I (EH)	<1%	23	38.3
	1-50%	24	40.0
	≥50%	13	21.7
Observer II (BBS)	<1%	16	26.7
	1-50%	29	48.3
	≥50%	15	25.0
Observer III (CS)	<1%	35	58.3
	1-50%	12	20.0
	≥50%	13	21.7
Observer IV (GU)	<1%	33	55.0
	1-50%	14	23.3
	≥50%	13	21.7

### Interpathologist Agreement of PD-L1 TPS

Comparison of the TPS values determined by four pathologists showed moderate agreement with Fleiss Kappa of 0.576 (Table 2). The statistical analysis of the three TPS categories [no/low expression (<1%), intermediate expression (1–49%) or high expression (≥50%)] demonstrated moderate agreement (Fleiss Kappa 0.477) in the “no/low” category, fair agreement (Fleiss Kappa 0.390) in the intermediate category, and almost perfect agreement (Fleiss Kappa 0.952) in the high category (Table 3).

**Table 2:** Interpathologist agreement of PD-L1 TPS.

**Overall Agreement**					
	**Kappa**	**Asymptotic**		**95% Confidence Interval**	
		**Std. Error**	**p**	**Lower**	**Upper**
Overall Agreement	0.576	0.038	**0.001**	0.574	0.579

**TPS:** Tumor Proportion Score

**Table 3:** Agreement on TPS categories.

**Agreement on TPS Categories**					
**TPS Category**	**Kappa**	**Asymptotic**		**95% Confidence Interval**	
		**Std. Error**	**p**	**Lower**	**Upper**
No/low expression	0.477	0.053	**0.001**	0.474	0.481
Intermediate expression	0.390	0.053	**0.001**	0.387	0.393
High expression	0.952	0.053	**0.001**	0.949	0.956

**TPS:** Tumor Proportion Score

### Histopathological Parameters

The histopathological examination revealed 37 cases of adenocarcinoma, 22 cases of squamous cell carcinoma, and 1 case of NSCLC, not otherwise specified (NOS). Tumor length range in the biopsy specimen was 1-41 millimeters, with a mean of 8.07 millimeters. Tumor ratio in the biopsy specimen was <50% in 31 cases and ≥50% in 29 cases. Necrosis was present in 16 cases. Tumor-infiltrating lymphocytes were ≥50% in 10 cases. A crush/squeeze artifact was present in 20 cases. Table 4 illustrates the histopathological features of the cases.

**Table 4:** Histopathological features of the cases.

**Histopathological Characteristics**				
** **		**Min-Max**	**Median**	**n (%)**
Histological type	Adenocarcinoma			37 (61.7)
	Squamous cell carcinoma			22 (36.7)
	NSCLC, NOS			1 (1.7)
Tumor length in biopsy (mm) (mean±sd)		1-41	5.50	8.07±7.068
Tumor ratio in biopsy	<50%			31 (51.7)
	≥50%			29 (48.3)
Necrosis	(-)			44 (73.3)
	(+)			16 (26.7)
TILs	<50%			50 (83.3)
	≥50%			10 (16.7)
Crush/squeeze artifact	(-)			40 (66.7)
	(+)			20 (33.3)

**NSCLC, NOS:** Non-small cell lung cancer, not otherwise specified; **TILs:** Tumor-infiltrating lymphocytes

### Relationship Between PD-L1 TPS Interpathologist Agreement and Histopathological Parameters

**Figure 2:**
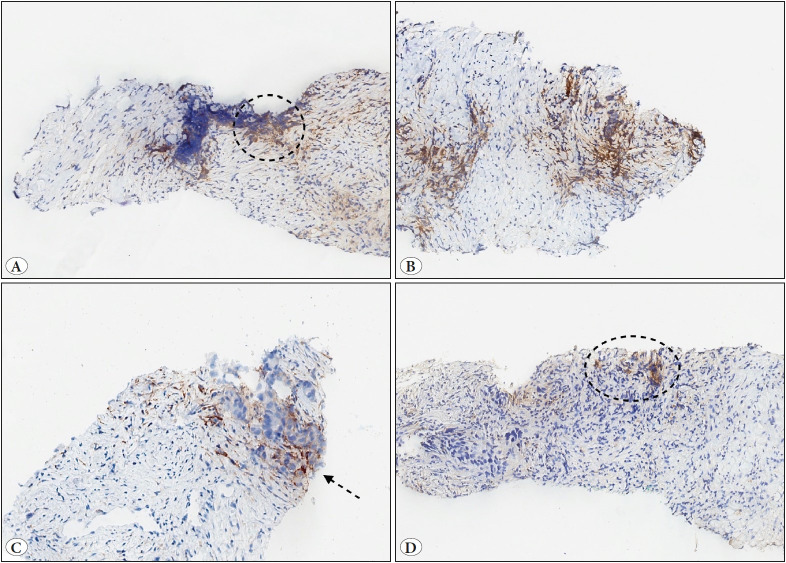
**A-D)** Examples of crush/squeeze artifact in PD-L1 immunohistochemistry studies (x100). **A)** The image illustrates some PDL1 staining cells in which the cell type (tumor cell/immune cell/stroma cell) cannot be determined clearly (circle). **B)** The details of the cell structure and the staining of PD-L1 cannot be clearly assessed due to the crush/squeeze artifact in this example. **C)** The tumor cells overlap with the immune cells at the edge of the biopsy specimen, making the identification of the correct cell types more challenging in PD-L1 staining (arrow). **D)** The area that shows staining with PD-L1 in this example contains crush artifacts that make it difficult to determine whether PD-L1 staining tumor cells are present among the immune cells (circle).

In order to investigate the relationship between TPS concordance and histopathological parameters, the study cases were divided into two groups: cases evaluated in the same TPS category by four pathologists (compatible group) and cases evaluated in a different TPS category by at least one pathologist (noncompatible group). As a result, 31 cases fell within the compatible group and 29 were within the noncompatible group. In terms of histological type, tumor size, tumor ratio, necrosis, and tumor-infiltrating lymphocytes, no statistically significant differences were found between the two groups. Compared to the compatible group, the noncompatible group had a significantly greater incidence of crush/squeeze artifacts (p<0.05) (Figure 2). Table 5 demonstrates the relationship between TPS concordance among pathologists and histopathological parameters.

**Table 5:** Relationship between TPS concordance among pathologists and histopathological parameters.

**Relationship of TPS concordance with histopathological parameters**						
		**Compatible group**		**Noncompatible group**		**p**
		**n (%)**	**Median**	**n (%)**	**Median**	
Histological type	Adenocarcinoma	20 (64.5)		17 (58.6)		0.507 **X²**
	SCC	10 (32.3)		12 (41.4)		
	NSCLC. NOS	1 (3.2)		0 (0.0)		
Tumor length (mm) (mean±sd)		8.88±8.043	6.50	7.14±5.765	5.00	0.422 **m**
Tumor ratio	<50%	13 (41.9)		18 (62.1)		0.119 **X²**
	≥50%	18 (58.1)		11 (37.9)		
Necrosis	(-)	20 (64.5)		24 (82.8)		0.110 **X²**
	(+)	11 (35.5)		5 (17.2)		
TILs	<50%	24 (77.4)		26 (89.7)		0.204 **X²**
	≥50%	7 (22.6)		3 (10.3)		
Crush/squeeze artifact	(-)	26 (83.9)		14 (48.3)		**0.003**** **X²**

**SCC:** Squamous cell carcinoma, **NSCLC, NOS:** Non-small cell lung cancer, not otherwise specified, **TILs:** Tumor-infiltrating lymphocytes, **m** Mannwhitney u test, **X²** Chi-square test

## DISCUSSION

The purpose of this study was to evaluate the agreement between pathologists for PD-L1 TPS in NSCLC needle core biopsy specimens. The results of this study demonstrated moderate agreement between four pathologists in PD-L1 TPS in tru-cut biopsy samples of NSCLCs. There was almost perfect agreement among pathologists for the 50% cut-off, but only fair agreement for the 1% cut-off, which is used to determine therapy eligibility. As a secondary objective, this study attempted to identify histopathological features that might be associated with interobserver variability. A statistically significant correlation did not exist between interpathologist agreement on PD-L1 scores and histological type, tumor length, tumor rate, necrosis and tumor-infiltrating lymphocytes. The crush/squeeze artifact, however, was found to significantly affect the interobserver agreement.

Prior studies investigating the interobserver agreement of TPS in NSCLC reported an overall substantial to almost perfect agreement, with only a few of the studies reporting moderate agreement (4–11) (Table 6). Compared to the previous studies, lower interobserver agreement regarding 1% cut-off was observed in our study. Considering that this study was conducted with four pathologists working in four different centers, it can be clearly seen how critical interpathologist compatibility in PDL1 scoring is in guiding patient treatment.

**Table 6:** Summary of prior studies on interobserver agreement of TPS in NSCLC.

**Summary of the studies on interpathologist agreement**				
**Study**	**Sample type**	**Number of samples**	**Number of pathologists**	**Interpathologist agreement (Kappa)§**
Rimm et al. (4)	Resection	90	13	0.537-0.749
Brunnström et al. (5)	TMA	55	7	0.712-0.948
Cooper et al. (6)	TMA	108	10	0.58-0.68
Scheel et al. (7)	TMA	21	10	0.73-0.89
Chang et al. (8)	Mixed	107 (22 resection, 66 NCB, 19 EBUS-TBNA)	27	0.633-0.834
De Marchi et al. (9)	Not disclosed	52	2	0.94
Butter et al. (10)	TMA	50	17	0.63
Yu et al. (11)	Resection	50	6	0.67-0.88
Our study	NCB	60	4	0.390-0.952

**TMA:** Tissue microarray, **NCB:** Needle core biopsy, **EBUS-TBNA:** Endobronchial ultrasound-guided transbronchial needle aspirate. § Different cut-off values for TPS were investigated in different studies. In this table, the lowest and highest kappa values specified in the study results are given.

The majority of the studies in the literature used resection samples or tissue microarrays (TMA), and only one included NCB specimens. To our knowledge, no study using only NCB materials has been published. There is a critical need to ensure high agreement between pathologists in PD-L1 evaluation in NCBs. This is because PD-L1 expression is assessed in the least invasive biopsy samples in the setting of advanced and metastatic disease, and this information guides treatment decisions. The only study in the literature that included NCB specimens investigated the interobserver reproducibility in a total of 107 NSCLC cases, consisting of 66 NCB specimens, 22 resection materials, and 19 endobronchial ultrasound-guided transbronchial needle aspirates (EBUS-TBNAs) (8). The authors reported higher agreement in resection and NCB specimens in comparison to EBUS-TBNA, but did not disclose a statistical comparison between resection materials and NCB specimens (kappa for NCB: 0.776, kappa for resection: 0.716) (8).

Considering the histopathological parameters that may be associated with high interpathologist variability, the results of our study have shown that there is no significant correlation between the pathologists’ agreement in TPS categories and the histological type of tumor, tumor length, tumor ratio in the biopsy specimen, presence of necrosis, and tumor-infiltrating lymphocytes. There was, however, a significant correlation between the presence of a crush/squeeze artifact in the biopsy and the rate of discordant TPS categories. In the literature, there is only one study that has investigated the relationship between NSCLC histological type and PD-L1 TPS interobserver agreement (8). This study indicated that interobserver agreement was influenced by histologic type, with the squamous cell carcinoma group showing slightly higher agreement than adenocarcinoma group (8). Our study is the first study to examine the effects of tumor necrosis, tumor-infiltrating lymphocytes, tumor length and percentage, and crush/squeeze artifact on interobserver variability.

A crush/squeeze artifact is typically observed at the periphery of the specimens, as a result of damage from crushing or squeezing by forceps or other surgical instruments (13). A crush/squeeze artifact presents difficulties in the assessment of cell types and the distinction of membranous staining from cytoplasmic staining. Membranous staining of other cell types, such as macrophages, stromal cells and necrotic tumor cells, and granular and cytoplasmic staining in tumor cells may be challenging to interpret in areas of crush/squeeze artifact. This may result in an incorrect assessment of the percentage of PD-L1 stained tumor cells. According to a recent study conducted on gastric, gastroesophageal junction and esophageal mucosal biopsy specimens, crush artifact was among the features associated with higher pathologist disagreements in PD-L1 scores (14). To our knowledge, no study has been published examining the relationship between crush artifact in NCB specimens from NSCLC and interpathologist agreement on PD-L1 scores.

A limitation of our study may be the small number of cases and participants. However, all our cases were sourced from and processed at one center, whereas the participating pathologists were affiliated with different institutions. The cases were representative of real clinical practice, and tissue processing and immunohistochemical staining followed the same standard procedure. Our study also has a limitation in that no relationship was established between the results and prognosis or treatment response. In order to provide a more comprehensive understanding of this issue, we are accelerating our studies on this subject in order to acquire data on treatment responses and prognoses.

In conclusion, our results indicate an overall moderate agreement between pathologists for PD-L1 TPS in NSCLC needle core biopsy materials. Significant correlation between the presence of a crush/squeeze artifact in the biopsy and the rate of discordant TPS categories was observed, indicating that PD-L1 assessment of needle core biopsies requires awareness of this artifact and meticulous examination.

## Conflict of Interest

The authors declare no conflict of interest.

## Funding

This study received no funding.
